# Níveis Elevados de Netrina-1 e IL-1β em Mulheres Idosas com SCA: Pior Prognóstico no Acompanhamento de Dois Anos

**DOI:** 10.36660/abc.20190035

**Published:** 2020-04-06

**Authors:** Paola Leocádio, Penélope Menta, Melissa Dias, Júlia Fraga, Alessandra Goulart, Itamar Santos, Paulo Lotufo, Isabela Bensenor, Jacqueline Alvarez-Leite

**Affiliations:** 1 Departamento de Bioquímica e ImunologiaUniversidade Federal de Minas GeraisBelo HorizonteMGBrasilDepartamento de Bioquímica e Imunologia - Universidade Federal de Minas Gerais, Belo Horizonte, MG – Brasil; 2 Centro de Pesquisa Clínica e EpidemiológicaHospital UniversitárioUniversidade de São PauloSão PauloSPBrasilCentro de Pesquisa Clínica e Epidemiológica do Hospital Universitário da Universidade de São Paulo, São Paulo, SP – Brasil; 3 Departamento de Clínica MédicaFaculdade de MedicinaUniversidade de São PauloSão PauloSPBrasilDepartamento de Clínica Médica da Faculdade de Medicina da Universidade de São Paulo, São Paulo, SP – Brasil

**Keywords:** Síndrome Coronariana Aguda/fisiopatologia, Nitrina-1, Interleucina- 1 beta, Remodelamento Atrial, Hipertensão, Diabetes Mellitus, Dislipidemias, Acidente Vascular Cerebral, Idoso, Mulheres

## Abstract

**Fundamento:**

Vários marcadores têm sido avaliados quanto a um potencial impacto nas decisões clínicas ou na predição de mortalidade na síndrome coronariana aguda (SCA), incluindo Netrina-1 e IL-1β.

**Objetivo:**

Examinamos o valor prognóstico de Netrina-1 e IL-1β em pacientes com SCA (2 anos de acompanhamento).

**Métodos:**

Avaliamos Netrina-1, IL-1β e outros fatores de risco em amostras de soro de 803 pacientes. Curvas de Kaplan-Meier e regressão de Cox foram usadas para análise de óbito por todas as causas, óbito por doenças cardiovasculares (DCV) e desfecho combinado de infarto agudo do miocárdio (IAM) fatal ou novo IAM não fatal, considerando p < 0,05.

**Resultados:**

Houve 115 óbitos por todas as causas, 78 óbitos por DCV e 67 eventos no desfecho combinado. Níveis de Netrina-1 acima da mediana (> 44,8 pg/mL) foram associados a pior prognóstico (óbito por todas as causas e por DCV) em mulheres idosas, mesmo após o ajuste do modelo (HR: 2,08, p = 0,038 e HR: 2,68, p = 0,036). Níveis de IL-1β acima da mediana (> 13,4 pg/mL) em mulheres idosas foram associados a risco aumentado para todos os desfechos após o ajuste (todas as causas - HR: 2,03, p = 0,031; DCV - HR: 3,01, p = 0,013; desfecho combinado - HR: 3,05, p = 0,029). Para homens, não foram observadas associações entre Netrina-1 ou IL-1β e os desfechos.

**Conclusão:**

Níveis séricos elevados de Netrina-1 e IL-1β mostraram associação significativa com pior prognóstico em idosas do sexo feminino. Eles podem ser úteis como indicadores prognósticos em SCA. (Arq Bras Cardiol. 2020; 114(3):507-514)

## Introdução

A doença arterial coronariana (DAC) é a principal causa de morte e anos de vida perdidos.^1^ Responsável pelo maior número de óbitos no Brasil, a DAC tem alta prevalência e prognóstico desfavorável.^2^ Apesar da redução na mortalidade por síndrome coronariana aguda (SCA) observada nas últimas décadas,^1^ estima-se que aproximadamente 14% dos pacientes que tiveram infarto agudo do miocárdio (IAM) morrerão em decorrência do mesmo.^3^ O risco de doença e morte é 1,5 a 15 vezes maior para pacientes que sobrevivem ao estágio agudo do IAM do que para a população geral.^3^ Daqueles que têm um primeiro IAM, aproximadamente 17% dos homens e 21% das mulheres com ≥ 45 anos terão um IAM recorrente ou DAC fatal dentro de cinco anos.^3^

A inflamação é um fator importante na fisiopatologia da SCA, bem como na remodelação cardíaca após IAM. Diversos marcadores foram avaliados quanto a um impacto potencial nas decisões clínicas ou na predição da mortalidade.^4^ Recentemente, moléculas de orientação neuronal, especialmente Netrina-1, foram identificadas como importantes moduladoras da aterosclerose, embora seu papel específico (protetor ou deletério) ainda seja controverso.^5-7^ A Netrina-1 faz parte de uma família de proteínas estruturalmente semelhantes às lamininas, que são componentes estruturais da membrana basal dos tecidos.^8^ O papel da Netrina-1 na doença cardiovascular (DCV) e na inflamação é uma área emergente de estudo.^5^

A interleucina-1 (IL-1) é um dos principais mediadores da coagulação induzida pela inflamação.^9^ A IL-1β é capaz de induzir a expressão de outras moléculas que favorecem o recrutamento de células inflamatórias para a lesão e injúria tecidual.^10^ Níveis elevados de IL-1 foram descritos no IAM.^11,12^ Recentemente, um ensaio clínico randomizado entre pessoas que sofreram um infarto do miocárdio mostrou que o canakinumab, um agente que bloqueia a IL-1β, reduziu a incidência de doença coronariana não fatal, eventos de acidente vascular cerebral não fatal e morte por DCV.^13^

Apesar da importância dessas duas moléculas na SCA, estudos avaliando o valor prognóstico das mesmas são escassos. Nosso objetivo foi avaliar o papel dessas moléculas como preditores de prognóstico em um acompanhamento de 2 anos.

## Métodos

### Desenho experimental

Os participantes fazem parte do estudo “ERICO” (Estratégia de Registro de Insuficiência Coronariana), descrito detalhadamente em trabalhos anteriores.^14,15^ Resumidamente, trata-se de um estudo de coorte prospectivo que incluiu indivíduos internados para tratamento de SCA no Hospital Universitário da Universidade de São Paulo (HU-USP), hospital de ensino com 260 leitos localizado no Butantã, São Paulo, Brasil, de fevereiro de 2009 a dezembro de 2013. O protocolo do estudo está de acordo com a Declaração de Helsinque. Este estudo foi aprovado pelo Comitê de Ética em Pesquisa (CEP-HU/USP 866/08) e todos os pacientes assinaram o Termo de Consentimento Livre e Esclarecido.

O IAM foi definido pela presença de sintomas compatíveis com isquemia miocárdica dentro de 24 horas da admissão hospitalar e nível de troponina I acima do valor do percentil 99 com um coeficiente de variação <10%. O infarto agudo do miocárdio com supradesnivelamento do segmento ST (IAMCSST) foi definido pelos critérios de IAM, além de: (a) a presença de supradesnivelamento do segmento ST persistente ≥1 mm em dois eletrocardiogramas contíguos (ECG de eletrodo) ou (b) novo (ou supostamente novo) bloqueio de ramo esquerdo (BRE). O infarto agudo do miocárdio sem supradesnivelamento do segmento ST (IAMSSST) foi definido pelos critérios para IAM mais ausência de supradesnivelamento do segmento ST persistente ≥ 1 mm em dois ECGs contíguos e de novo ou supostamente novo BRE. A angina instável (AI) foi definida como a presença de sintomas compatíveis com isquemia miocárdica nas últimas 24 horas, ausência de diagnóstico de IAM e pelo menos um dos cinco critérios a seguir: (a) história prévia de DAC; (b) estratificação positiva de doença isquêmica invasiva ou não invasiva do coração; (c) alterações dinâmicas ou evolutivas do ECG; (D) troponina I > 0,4 ng/mL (assegurando níveis de troponina I acima do percentil 99, independentemente do kit utilizado) ou (e) concordância no diagnóstico de AI entre dois médicos independentes.

### Coleta de dados e desfechos

Após seis meses e anualmente por dois anos após a admissão hospitalar, todos os indivíduos foram contatados por telefone para atualizar as informações do estado vital, incluindo desfechos cardiovasculares fatais e não fatais. Sempre que um participante relatou um possível novo evento de IAM, novos procedimentos de investigação foram iniciados para confirmar o evento.

Os desfechos avaliados foram: óbito por todas as causas, óbito por DCV e o desfecho combinado (IAM fatal ou novo IAM não fatal). A estratégia para coletar e classificar os dados de mortalidade, incluindo a busca por registros oficiais de óbitos, foi detalhada em estudo anterior.^15^ Nos casos em que não foi possível determinar a causa da morte, os dados foram censurados para todos os desfechos, com exceção da morte por todas as causas.

Durante a fase hospitalar, entrevistadores treinados coletaram dados relacionados às características sociodemográficas, fatores de risco cardiovascular e medicação, conforme descrito anteriormente.^15^ Amostras de sangue foram coletadas dentro de 24 horas da admissão. Análises de glicose plasmática, triglicérides, colesterol total e HDL foram realizadas no HU-USP. O colesterol LDL foi calculado pela equação de Friedewald.^16^ As concentrações de Netrina-1 e IL-1β na admissão foram avaliadas por *Enzyme-Linked Immunosorbent Assay* (ELISA), seguindo as instruções do kit (Netrina-1: SEB827HU; USCN Life Science Inc., Wuhan, China e IL-1β: 88- 7010-88 eBioscience Inc., San Diego, CA, EUA). Os pacientes foram classificados de acordo com as concentrações de Netrina-1 e IL-1β nos grupos “baixa” e “alta”, se sua concentração estivesse abaixo ou acima da mediana.

### Análise estatística

Os dados foram avaliados quanto à normalidade usando o teste de Kolmogorov-Smirnov. Os testes qui-quadrado e Mann-Whitney (todas as variáveis contínuas apresentaram distribuição não-paramétrica) foram utilizados para comparar os grupos. Os valores foram expressos como mediana (intervalo interquartil) ou n (%). Curvas de Kaplan-Meier foram utilizadas, e o teste log-rank foi utilizado para avaliar a diferença entre os grupos. As estimativas de risco (razões de risco com seus respectivos intervalos de confiança de 95%) para os eventos foram calculadas usando regressão de Cox. Além de Netrina-1 e IL-1β, as seguintes variáveis foram usadas para construir modelos: idade, tipo de SCA, diabetes, hipertensão e dislipidemia. Um valor de p < 0,05 foi considerado significativo.

Os programas SPSS (IBM SPSS Statistics para Windows, versão 22.0, Armonk, NY: IBM Corp.) e GraphPad Prism (versão 5.01 para Windows, San Diego, Califórnia: GraphPad Software) foram usados para realizar as análises.

## Resultados

Um total de 803 pacientes foram incluídos neste estudo, incluindo 333 mulheres e 470 homens. Comparando as principais características dos grupos de homens e mulheres, observamos que as mulheres eram mais velhas e apresentavam maior concentração de HDL que os homens. As mulheres também foram mais afetadas pela hipertensão, diabetes e dislipidemia (Tabela 1). O tipo mais frequente de SCA entre os grupos foi o IAMSSST (cerca de 40% dos casos), seguido por AI e IAMCSST, que tiveram frequência semelhante (cerca de 30% cada).

**Tabela 1:** – Características gerais na fase hospitalar em homens e mulheres

Parâmetro	Homens (n = 470)	Mulheres (n = 333)	p
Idade	60 (52 – 71)	65 (56 – 76)	< 0,0001
IMC	26,8 (23,8 – 29,6)	26,8 (24,0 – 30,9)	0,128
**Tipo SCA**			**0,027**
IAMSSST	191 (40,6)	142 (42,6)	
IAMCSST	147 (31,3)	77 (23,1)	
AI	132 (28,1)	114 (34,2)	
**Tabagismo**			
Atual	141 (31,3)	85 (27,2)	< 0,0001
Passado	198 (43,9)	82 (26,3)	
Nunca	112 (24,8)	145 (46,5)	
Hipertensão	339 (73,7)	267 (80,9)	0,018
Diabetes	156 (34,7)	148 (45,0)	0,004
Dislipidemia	197 (48,0)	181 (60,7)	0,001
Glicose	125,0 (101,0 – 157,0)	124,0 (103,0 – 175,0)	0,652
Triglicerídeos	132,0 (94,0 – 190,3)	126,0 (97,0 – 183,0)	0,685
Colesterol total	171,5 (141,0 – 205,0)	170,0 (139,0 – 204,0)	0,720
Colesterol HDL	35,0 (30,0 – 44,0)	39,0 (32,0 – 46,5)	< 0,0001
Colesterol LDL	102,5 (77,0 – 134,3)	99,0 (77,0 – 124,3)	0,386
Netrina-1	44,8 (34,2 – 65,8)	44,8 (34,8 – 62,8)	0,813
IL-1β	15,1 (7,4 - 28,8)	13,8 (7,1 – 29,7)	0,536

Mediana (intervalo interquartil) ou n (%). AI: angina instável. HDL: lipoproteína de alta densidade. IAMCSST: infarto do miocárdio com supradesnivealmento do segmento ST. IAMSSST: infarto do miocárdio sem supradesnivealmento do segmento ST. IL-1β: interleucina - 1beta. IMC: índice de massa corporal em kg/m^2^. LDL: lipoproteína de baixa densidade. SCA: síndrome coronariana aguda. Dados de glicose plasmática, triglicerídeos, colesterol total, HDL e LDL são apresentados em mg/dL. Netrina-1 e IL-1β são apresentados em pg/mL. Teste de Mann-Whitney ou teste de qui-quadrado. p-valor comparando homens e mulheres.

Durante os dois anos de acompanhamento, houve 115 mortes por todas as causas (65 homens e 50 mulheres), incluindo 78 mortes (67,8%) por causas cardiovasculares. Também identificamos 67 casos de IAM (fatal ou não fatal) neste mesmo seguimento. Como a idade é um fator importante envolvido na taxa de mortalidade, analisamos separadamente em mulheres e homens jovens e com mais de 60 anos.

Para avaliar um possível papel de Netrina-1 e IL-1β como marcadores prognósticos, comparamos a frequência de SCA nos pacientes com níveis de Netrina-1 e IL-1β acima e abaixo da respectiva mediana. Não houve associações entre os níveis de Netrina-1 e IL-1β e todos os desfechos para homens independentemente de sua idade (dados não mostrados). Por esse motivo, focamos nossa investigação no grupo de mulheres (333 pacientes). As principais características do grupo de mulheres (jovens e idosas) são mostradas na Tabela 2.

**Tabela 2:** – Características gerais na fase hospitalar em mulheres jovens (< 60 anos) e idosas (> 60 anos)

Parâmetro	Total (n = 333)	Jovens (< 60 anos) (n = 111)	Idosas (> 60 anos) (n = 222)	p
IMC	26,8 (24,0 – 30,9)	26,8 (24,5 - 31,1)	26,7 (23,8 – 30,8)	0,532
**Tipo SCA**				**0,031**
IAMSSST	142 (42,6)	40 (36,0)	102 (45,9)	
IAMCSST	77 (23,1)	35 (31,5)	42 (18,9)	
AI	114 (34,2)	36 (32,4)	78 (35,1)	
**Tabagismo**				**< 0,0001**
Atual	85 (27,2)	47 (44,3)	38 (18,4)	
Passado	82 (26,3)	28 (26,4)	54 (26,2)	
Nunca	145 (46,5)	31 (29,2)	114 (55,3)	
Hipertensão	267 (80,9)	79 (72,5)	188 (85,1)	0,006
Diabetes	148 (45,0)	39 (35,8)	109 (49,5)	0,018
Dislipidemia	181 (60,7)	48 (48,5)	133 (66,8)	0,002
Glicose	124,0 (103,0 – 175,0)	121,0 (103,0 – 163,0)	128,0 (104,0 – 180,0)	0,381
Triglicerídeos	126,0 (97,0 – 183,0)	151,0 (97,0 – 201,0)	122,0 (93,5 – 174,0)	0,080
Colesterol total	170,0 (139,0 – 204,0)	185,0 (157,5 – 215,3)	161,0 (134,0 – 196,0)	0,002
Colesterol HDL	39,0 (32,0 – 46,5)	36,0 (32,0 – 45,0)	41,0 (33,0 – 47,2)	0,065
Colesterol LDL	99,0 (77,0 – 124,3)	114,0 (89,0 – 134,0)	93,0 (72,0 – 118,0)	0,0004
Netrina-1	44,8 (34,8 – 62,8)	44,8 (33,8 – 65,8)	44,8 (34,7 – 65,0)	0,861
IL-1β	13,8 (7,1 – 29,7)	15,5 (7,9 – 49,7)	13,4 (7,1 – 24,1)	0,037

Mediana (intervalo interquartil) ou n (%). AI: angina instável. HDL: lipoproteína de alta densidade. IAMCSST: infarto do miocárdio com supradesnivealmento do segmento ST. IAMSSST: infarto do miocárdio sem supradesnivealmento do segmento ST. IL-1β: interleucina - 1beta. IMC: índice de massa corporal em kg/m^2^. LDL: lipoproteína de baixa densidade. SCA: síndrome coronariana aguda. Dados de glicose plasmática, triglicerídeos, colesterol total, HDL e LDL são apresentados em mg/dL. Netrina-1 e IL-1β são apresentados em pg/mL. Teste de Mann-Whitney ou teste de qui-quadrado.

Na admissão, as mulheres apresentaram valores semelhantes de índice de massa corporal, concentrações séricas de glicose, triglicerídeos e colesterol HDL, independentemente do grupo etário. A frequência de fatores de risco como hipertensão, dislipidemia e diabetes foi maior em mulheres idosas. No entanto, os níveis de colesterol LDL foram menores nas idosas. Fumantes atuais foram mais frequentes em mulheres jovens, enquanto mais de 50% das mulheres idosas nunca fumou (Tabela 2). Não encontramos diferenças na mediana de Netrina-1 entre os grupos etários. No entanto, a mediana de IL-1β foi maior no grupo mais jovem.

As associações entre Netrina-1 ou IL1-β baixa e alta e os desfechos, de acordo com a faixa etária, foram apresentadas na Tabela 3 e na Tabela 4, respectivamente. O número de mortes por todas as causas foi muito baixo (3 mortes) nas mulheres com menos de 60 anos e apenas 6 casos do desfecho combinado, dificultando análises confiáveis neste grupo. No entanto, no grupo mais velho (> 60 anos), encontramos associações entre níveis elevados de Netrina-1 e mortes por todas as causas e causas cardiovasculares. Uma associação entre IL-1β alta e morte por DCV também foi encontrada entre as mulheres idosas (p = 0,034).

**Tabela 3:** – Desfechos de acordo com os níveis de Netrina-1 em mulheres

	Mulheres - Total			< 60 anos			> 60 anos		
Acompanhamento de 2 anos	Netrina-1 baixa	Netrina-1 alta	p	Netrina-1 baixa	Netrina-1 alta	p	Netrina-1 baixa	Netrina-1 alta	p
Óbito por todas as causas	18 (36,0)	32 (64,0)	0,021	2 (66,7)	1 (33,3)	0,612	16 (34,0)	31 (66,0)	0,011
Óbito por DCV	12 (35,3)	22 (64,7)	0,052	2 (66,7)	1 (33,3)	0,612	10 (32,3)	21 (67,7)	0,029
IAM fatal ou novo IAM não fatal	11 (39,3)	17 (60,7)	0,193	4 (66,7)	2 (33,3)	0,467	7 (31,8)	15 (68,2)	0,066

n (%). DCV: doenças cardiovasculares. IAM: infarto agudo do miocárdio. Teste do qui-quadrado.

**Tabela 4:** – Desfechos de acordo com os níveis de IL-1β em mulheres

	Mulheres - Total			< 60 anos			> 60 anos		
Acompanhamento de 2 anos	IL-1β baixa	IL-1β alta	p	IL-1β baixa	IL-1β alta	p	IL-1β baixa	IL-1β alta	p
Óbito por todas as causas	21 (42,0)	29 (58,0)	0,221	0 (0,0)	3 (100,0)	0,118	19 (40,4)	28 (59,6)	0,140
Óbito por DCV	12 (35,3)	22 (64,7)	0,071	0 (0,0)	3 (100,0)	0,118	10 (32,3)	21 (67,7)	0,034
IAM fatal ou novo IAM não fatal	10 (35,7)	18 (64,3)	0,117	2 (33,3)	4 (66,7)	0,438	7 (31,8)	15 (68,2)	0,075

n (%). DCV: doenças cardiovasculares. IAM: infarto agudo do miocárdio. IL-1β: Interleucina - 1beta. Teste do qui-quadrado.

**Figura 1:**
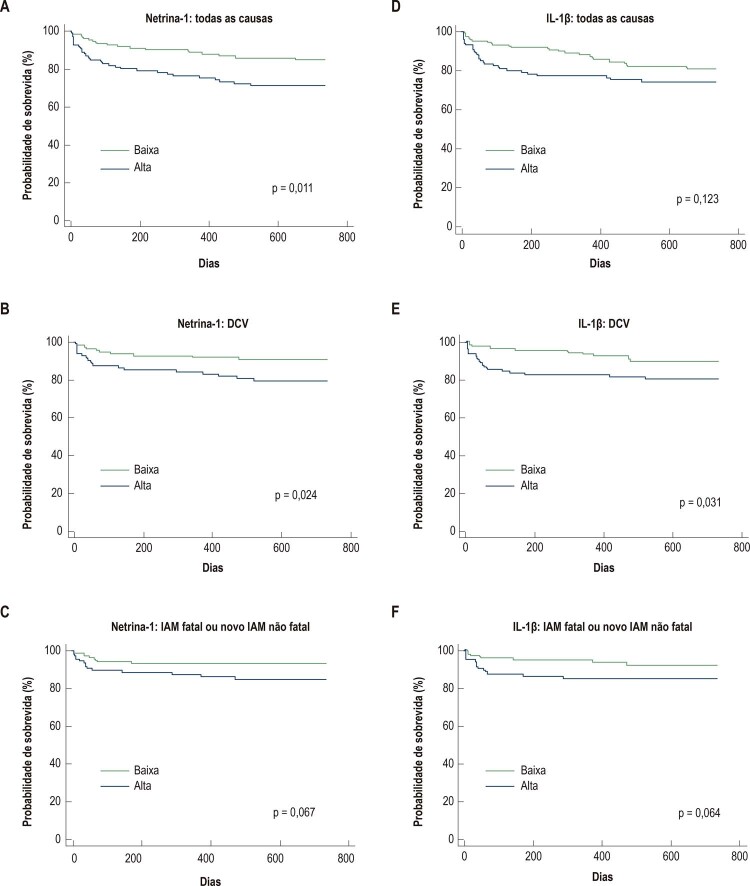
– Curvas de Kaplan-Meier para Netrina-1 (A – C) e IL-1β (D – F) em mulheres idosas (> 60 anos) no acompanhamento de dois anos. IL-1β: Interleucina - 1beta; DCV: doença cardiovascular; IAM: infarto agudo do miocárdio.

Os dados mostrando um pior prognóstico em mulheres idosas com níveis elevados de Netrina-1 e IL-1β foram confirmados pelas curvas de Kaplan-Meier. Níveis elevados de Netrina-1 resultaram em uma menor taxa de sobrevivência quando se considera a mortalidade por todas as causas (p = 0,011, Figura 1A) e também considerando apenas mortes por DCV (p = 0,024, Figura 1B). Houve tendência de associação a IAM fatal ou novo IAM não fatal (p = 0,067, Figura 1C). Níveis elevados de IL-1β também mostraram uma menor taxa de sobrevivência quando consideradas mortes por DCV (p = 0,031, Figura 1E), com tendência a associação com o desfecho combinado (p = 0,064, Figura 1F).

A análise das razões de risco (Tabela 5) mostrou um aumento no risco de morte por todas as causas para o grupo Netrina-1 alta, que permaneceu significativo no modelo ajustado. Os mesmos resultados foram observados para o risco de morte por DCV.

**Tabela 5:** – Razão de risco (HR) para os níveis de Netrina-1 e IL-1β e óbito por todas as causas, óbito por doenças cardiovasculares e desfecho combinado de IAM fatal ou novo IAM não fatal em mulheres idosas (> 60 anos)

	Óbito por todas as causas		Óbito por DCV		IAM fatal ou novo IAM não fatal	
	HR (IC 95%)	p	HR (IC 95%)	p	HR (IC 95%)	p
	**Netrina-1**					
Crua	2,15 (1,17 – 3,93)	0,013	2,31 (1,09 – 4,92)	0,029	2,25 (0,92 – 5,54)	0,075
Ajustada *	2,08 (1,04 – 4,16)	0,038	2,68 (1,06 – 6,74)	0,036	1,82 (0,65 – 5,07)	0,247
	**IL-1β**					
Crua	1,57 (0,87 – 2,81)	0,127	2,23 (1,05 – 4,75)	0,036	2,27 (0,92 – 5,58)	0,073
Ajustada *	2,03 (1,06 – 3,89)	0,031	3,01 (1,26 – 7,17)	0,013	3,05 (1,12 – 8,32)	0,029

HR: razão de risco. IAM: infarto agudo do miocárdio. IC: intervalo de confiança. IL-1β: Interleucina - 1beta. * Ajustado por idade, tipo de síndrome coronariana aguda, diabetes, hipertensão e dislipidemia.

Considerando os níveis elevados de IL-1β, não encontramos HR significativa no modelo cru para mortalidade por todas as causas e para o desfecho combinado (Tabela 5). No entanto, foi observada HR significativa para mortalidade por todas as causas e desfecho combinado no modelo ajustado. Observamos também um risco aumentado de morte por causas cardiovasculares para o grupo IL-1β alta, mesmo após o ajuste do modelo.

## Discussão

Este trabalho é pioneiro na avaliação do valor prognóstico da Netrina-1 na SCA e apresenta novas informações sobre o valor prognóstico da IL-1β nessa condição.

Em nosso estudo, observamos uma associação entre níveis elevados de Netrina-1 e pior prognóstico quando foram analisados óbito por todas as causas e por DCV em mulheres idosas.

A Netrina-1 plasmática é um biomarcador diagnóstico de muitos tipos de câncer.^17-19^ Verificou-se que a maior expressão gênica ou concentração de Netrina-1 nesses tecidos é associada a um pior prognóstico, provavelmente relacionado aos efeitos antiapoptóticos e angiogênicos da molécula. No entanto, o papel da Netrina-1 na aterosclerose e na remodelação cardíaca após o IAM ainda é controverso. Foi descrito que Netrina-1 poderia promover ou proteger contra a aterosclerose, dependendo das condições ambientais.^20^ Níveis endógenos reduzidos de Netrina-1 também podem levar a efeitos deletérios, uma vez que fatores pró-aterogênicos podem reduzir a expressão dessa molécula.^20^ Em modelos de IAM, a administração de netrina-1 reduziu a gravidade da lesão miocárdica quando comparada aos controles não suplementados.^21,22^

Em nosso estudo, é possível que o grupo “Netrina-1 alta" seja composto por pacientes que tiveram um evento mais grave de SCA. Nossa hipótese é que o nível de Netrina-1 aumenta em casos mais graves de SCA. Esta hipótese baseia-se em estudos que indicam que a expressão de Netrina-1 é induzida após lesão celular e pode ser usada como um biomarcador para danos ou doenças de órgãos,^23^ como visto na cirurgia cardíaca.^24^ Além disso, a hipóxia, uma condição intimamente ligada à aterosclerose e à SCA, também é um indutor da expressão da Netrina-1.^25^ Ainda, Van Gils et al.^6^ observaram aumento da expressão da molécula em macrófagos carregados de colesterol promovendo a retenção dessas células *in vitro*, o que poderia contribuir para uma evolução mais rápida da placa aterosclerótica e, consequentemente, aumentar a chance de formação de trombo e ocorrência de infarto.

Esses fatores nos levam a acreditar que o maior número de desfechos desfavoráveis, bem como o maior risco de morte por todas as causas e por DCV observados em mulheres idosas com níveis mais elevados de Netrina-1 estão associados à gravidade do evento e ao maior grau de inflamação (como sugerido pelos altos níveis de IL-1β) que podem ter contribuído para um pior prognóstico.

Em relação a IL-1β, níveis elevados desta e de outras citocinas pró-inflamatórias já foram identificados em pacientes com SCA.^26^ No entanto, poucos estudos foram direcionados ao valor prognóstico dessa citocina.^27,28^ Corroborando nossos resultados, esses estudos sugerem que níveis mais altos de IL-1β foram observados em pacientes com SCA que sofreram novos eventos durante o acompanhamento.

A IL-1β é capaz de aumentar a expressão de moléculas que contribuem para a ruptura da placa e formação de trombos, culminando na ocorrência de SCA.^10,29^ Assim, níveis mais elevados de IL-1β sérica poderiam refletir inflamação exacerbada, favorecendo a ocorrência de complicações cardiovasculares.

Níveis mais altos de IL-1β sugerem uma inflamação exacerbada que pode prejudicar o remodelamento cardíaco. A remodelação adversa após o IAM é a base estrutural da insuficiência cardíaca isquêmica. Embora quantidades adequadas de IL-1β e outras citocinas inflamatórias sejam essenciais na fase inicial do remodelamento, a diminuição dos níveis de citocinas é necessária para promover uma cicatrização efetiva.^30^ Foi descrito que níveis elevados de IL-1β até dois meses após o infarto em pacientes com IAMCSST estavam fortemente associados à piora da função cardíaca após um ano de acompanhamento.^31^ Além disso, a citocina foi um forte preditor de hipertrofia ventricular esquerda, importante na predição da morbidade e mortalidade cardiovascular.^31^

Vários fatores podem ajudar a entender a ausência de associação com pior prognóstico encontrada em homens e mulheres mais jovens. Os tecidos cardíacos e vasculares são influenciados por hormônios como estrógeno e testosterona, variando de acordo com sexo e idade.^32,33^ Mulheres mais velhas têm maior massa do ventrículo esquerdo do que homens, devido a fatores que indicam menor capacidade arterial, como redução da espessura da parede da carotída.^33^

A relação entre citocinas inflamatórias e gênero ainda não foi elucidada, embora diferenças na concentração sejam observadas na literatura.^32^ Estudos indicam que a concentração de IL-β é maior no sexo masculino.^33,34^ Além disso, níveis de citocinas estão inversamente relacionados à idade, como visto em nosso trabalho e na literatura.^35^ A literatura não fornece dados sobre diferenças de sexo para Netrina-1. Como não observamos diferenças estatísticas entre os sexos nos níveis dos marcadores, nossa hipótese é de que no sexo feminino os níveis destes eram mais altos que os normais. Esse possível aumento pode estar relacionado à maior frequência de fatores de risco cardiovasculares neste grupo no presente estudo, fatores que podem levar a um aumento dos níveis de marcadores inflamatórios.^36^ No entanto, mesmo após o ajuste desses fatores, níveis elevados de Netrina 1 e IL-1β permaneceram associados a pior prognóstico, demonstrando que esses marcadores inflamatórios estão independentemente associados a pior prognóstico e podem estar relacionados à redução da capacidade arterial já observada em mulheres idosas, quando comparados aos homens.

Apontamos como limitações do estudo sua característica unicêntrica e a ausência de um grupo controle. Ainda, não coletamos dados do período pré-evento, o que nos permitiria determinar a variação na concentração do marcador após a SCA.

## Conclusão

Níveis elevados de Netrina-1 e IL-1β estão associados a pior prognóstico em mulheres idosas com SCA. O mecanismo para tal associação pode estar relacionado à manutenção da inflamação e remodelação cardíaca adversa, propiciando novos eventos cardiovasculares.

## References

[1] . Collaborators GDaH. Global, regional, and national disability-adjusted life-years (DALYs) for 315 diseases and injuries and healthy life expectancy (HALE), 1990-2015: a systematic analysis for the Global Burden of Disease Study 2015. Lancet. 2016;388(10053):1603-58. 10.1016/S0140-6736(16)31460-X PMC5388857 27733283

[2] . Ribeiro AL, Duncan BB, Brant LC, Lotufo PA, Mill JG, Barreto SM. Cardiovascular Health in Brazil: Trends and Perspectives. Circulation. 2016;133(4):422-33. 10.1161/CIRCULATIONAHA.114.008727 26811272

[3] . Benjamin EJ, Blaha MJ, Chiuve SE, Cushman M, Das SR, Deo R, et al. Heart Disease and Stroke Statistics-2017 Update: A Report From the American Heart Association. Circulation. 2017;135(10):e146-e603. 10.1161/CIR.0000000000000485 PMC5408160 28122885

[4] . Basra SS, Virani SS, Paniagua D, Kar B, Jneid H. Acute Coronary Syndromes: Unstable Angina and Non-ST Elevation Myocardial Infarction. Heart Fail Clin. 2016;12(1):31-48. 10.1016/j.hfc.2015.08.004 26567973

[5] . Layne K, Ferro A, Passacquale G. Netrin-1 as a novel therapeutic target in cardiovascular disease: to activate or inhibit? Cardiovasc Res. 2015;107(4):410-9. 10.1093/cvr/cvv201 26209250

[6] . van Gils JM, Derby MC, Fernandes LR, Ramkhelawon B, Ray TD, Rayner KJ, et al. The neuroimmune guidance cue netrin-1 promotes atherosclerosis by inhibiting the emigration of macrophages from plaques. Nat Immunol. 2012;13(2):136-43. 10.1038/ni.2205 PMC3262880 22231519

[7] . Bouhidel JO, Wang P, Siu KL, Li H, Youn JY, Cai H. Netrin-1 improves post-injury cardiac function in vivo via DCC/NO-dependent preservation of mitochondrial integrity, while attenuating autophagy. Biochim Biophys Acta. 2015;1852(2):277-89. 10.1016/j.bbadis.2014.06.005 PMC4262720 24928309

[8] . Cirulli V, Yebra M. Netrins: beyond the brain. Nat Rev Mol Cell Biol. 2007;8(4):296-306. 10.1038/nrm2142 17356579

[9] . Libby P, Tabas I, Fredman G, Fisher EA. Inflammation and its resolution as determinants of acute coronary syndromes. Circ Res. 2014;114(12):1867-79. 10.1161/CIRCRESAHA.114.302699 PMC4078767 24902971

[10] . Frangogiannis NG. The immune system and cardiac repair. Pharmacol Res. 2008;58(2):88-111. 10.1016/j.phrs.2008.06.007 PMC2642482 18620057

[11] . Dewald O, Ren G, Duerr GD, Zoerlein M, Klemm C, Gersch C, et al. Of mice and dogs: species-specific differences in the inflammatory response following myocardial infarction. Am J Pathol. 2004;164(2):665-77. 10.1016/S0002-9440(10)63154-9 PMC1602262 14742270

[12] . Ertl G, Frantz S. Healing after myocardial infarction. Cardiovasc Res. 2005;66(1):22-32. 10.1016/j.cardiores.2005.01.011 15769445

[13] . Ridker PM, Everett BM, Thuren T, MacFadyen JG, Chang WH, Ballantyne C, et al. Antiinflammatory Therapy with Canakinumab for Atherosclerotic Disease. N Engl J Med. 2017;377(12):1119-31. 10.1056/NEJMoa1707914 28845751

[14] . Santos IS, Goulart AC, Brandão RM, Santos RC, Bittencourt MS, Sitnik D, et al. One-year Mortality after an Acute Coronary Event and its Clinical Predictors: The ERICO Study. Arq Bras Cardiol. 2015;105(1):53-64. 10.5935/abc.20150044 PMC4523288 25993485

[15] . Goulart AC, Santos IS, Sitnik D, Staniak HL, Fedeli LM, Pastore CA, et al. Design and baseline characteristics of a coronary heart disease prospective cohort: two-year experience from the strategy of registry of acute coronary syndrome study (ERICO study). Clinics (Sao Paulo). 2013;68(3):431-4. 10.6061/clinics/2013(03)RC02 PMC3611751 23644870

[16] . Friedewald WT, Levy RI, Fredrickson DS. Estimation of the concentration of low-density lipoprotein cholesterol in plasma, without use of the preparative ultracentrifuge. Clin Chem. 1972;18(6):499-502. 4337382

[17] . Ramesh G, Berg A, Jayakumar C. Plasma netrin-1 is a diagnostic biomarker of human cancers. Biomarkers. 2011;16(2):172-80. 10.3109/1354750X.2010.541564 PMC3143477 21303223

[18] . Link BC, Reichelt U, Schreiber M, Kaifi JT, Wachowiak R, Bogoevski D, et al. Prognostic implications of netrin-1 expression and its receptors in patients with adenocarcinoma of the pancreas. Ann Surg Oncol. 2007;14(9):2591-9. 10.1245/s10434-007-9469-6 17549567

[19] . Harter PN, Zinke J, Scholz A, Tichy J, Zachskorn C, Kvasnicka HM, et al. Netrin-1 expression is an independent prognostic factor for poor patient survival in brain metastases. PLoS One. 2014;9(3):e92311. 10.1371/journal.pone.0092311 PMC3960244 24647424

[20] . van Gils JM, Ramkhelawon B, Fernandes L, Stewart MC, Guo L, Seibert T, et al. Endothelial expression of guidance cues in vessel wall homeostasis dysregulation under proatherosclerotic conditions. Arterioscler Thromb Vasc Biol. 2013;33(5):911-9. 10.1161/ATVBAHA.112.301155 PMC3647028 23430612

[21] . Zhang J, Cai H. Netrin-1 prevents ischemia/reperfusion-induced myocardial infarction via a DCC/ERK1/2/eNOS s1177/NO/DCC feed-forward mechanism. J Mol Cell Cardiol. 2010;48(6):1060-70. 10.1016/j.yjmcc.2009.11.020 PMC2866819 20004665

[22] . Mao X, Xing H, Mao A, Jiang H, Cheng L, Liu Y, et al. Netrin-1 attenuates cardiac ischemia reperfusion injury and generates alternatively activated macrophages. Inflammation. 2014;37(2):573-80. 10.1007/s10753-013-9771-3 24234226

[23] . Reeves WB, Kwon O, Ramesh G. Netrin-1 and kidney injury. II. Netrin-1 is an early biomarker of acute kidney injury. Am J Physiol Renal Physiol. 2008;294(4):F731-8. 10.1152/ajprenal.00507.2007 18234954

[24] . Ramesh G, Krawczeski CD, Woo JG, Wang Y, Devarajan P. Urinary netrin-1 is an early predictive biomarker of acute kidney injury after cardiac surgery. Clin J Am Soc Nephrol. 2010;5(3):395-401. 10.2215/CJN.05140709 PMC2827571 20007677

[25] . Ramkhelawon B, Yang Y, van Gils JM, Hewing B, Rayner KJ, Parathath S, et al. Hypoxia induces netrin-1 and Unc5b in atherosclerotic plaques: mechanism for macrophage retention and survival. Arterioscler Thromb Vasc Biol. 2013;33(6):1180-8. 10.1161/ATVBAHA.112.301008 PMC3793633 23599441

[26] . Ozeren A, Aydin M, Tokac M, Demircan N, Unalacak M, Gurel A, et al. Levels of serum IL-1beta, IL-2, IL-8 and tumor necrosis factor-alpha in patients with unstable angina pectoris. Mediators Inflamm. 2003;12(6):361-5. 10.1080/09629350310001633360 PMC1781634 14668096

[27] . Kilic T, Ural D, Ural E, Yumuk Z, Agacdiken A, Sahin T, et al. Relation between proinflammatory to anti-inflammatory cytokine ratios and long-term prognosis in patients with non-ST elevation acute coronary syndrome. Heart. 2006;92(8):1041-6. 10.1136/hrt.2005.080382 PMC1861097 16547209

[28] . Correia LC, Andrade BB, Borges VM, Clarêncio J, Bittencourt AP, Freitas R, et al. Prognostic value of cytokines and chemokines in addition to the GRACE Score in non-ST-elevation acute coronary syndromes. Clin Chim Acta. 2010;411(7-8):540-5. 10.1016/j.cca.2010.01.011 20083097

[29] . Prabhu SD, Frangogiannis NG. The Biological Basis for Cardiac Repair After Myocardial Infarction: From Inflammation to Fibrosis. Circ Res. 2016;119(1):91-112. 10.1161/CIRCRESAHA.116.303577 PMC4922528 27340270

[30] . Frantz S, Bauersachs J, Ertl G. Post-infarct remodelling: contribution of wound healing and inflammation. Cardiovasc Res. 2009;81(3):474-81. 10.1093/cvr/cvn292 PMC2639128 18977766

[31] . Orn S, Ueland T, Manhenke C, Sandanger O, Godang K, Yndestad A, et al. Increased interleukin-1beta levels are associated with left ventricular hypertrophy and remodelling following acute ST segment elevation myocardial infarction treated by primary percutaneous coronary intervention. J Intern Med. 2012;272(3):267-76. 10.1111/j.1365-2796.2012.02517.x 22243053

[32] . Bourgeois M M, Richards I S. Gender-specific differences in the urinary expression of aldosterone, IL-1α and IL-1β. Biomarkers Med. 2010;4(6):843–47. 10.2217/bmm.10.102 21133705

[33] . Haroon J, Foureaux G, Martins A S, Ferreira A J, Reis A M, Javed Q. Gender differences in normal left ventricle of adult FVB/N mice due to variation in interleukins and natriuretic peptides expression levels. Cytokine. 2015;71(1):54-59. 10.1016/j.cyto.2014.08.008 25226444

[34] . Lyngdoh T, Marques-Vidal P, Paccaud F, Preisig M, Waeber G, Bochud M, et al. Elevated serum uric acid is associated with high circulating inflammatory cytokines in the population- based CoLaus study. PLoS ONE. 2011;6(5):e19901. 10.1371/journal.pone.0019901 PMC3098830 21625475

[35] . Marques-Vidal P, Bochud M, Bastardot F, Luscher T, Ferrero F, Gaspoz J, et al. Levels and determinants of inflammatory biomarkers in a swiss population-based sample (CoLaus study). PLoS ONE. 2011;6(6):e21002. 10.1371/journal.pone.0021002 PMC3111463 21695270

[36] . Yim J, Kim G, Lee B, Kang E S, Cha B, Kim J, et al. Relationship between circulating netrin-1 concentration, impaired fasting glucose, and newly diagnosed type 2 diabetes. Front Endocrinol. 2018;9(691). 10.3389/fendo.2018.00691 PMC6265472 30532735

