# Supplementary material for: Níveis Elevados de Netrina-1 e IL-1β em Mulheres Idosas com SCA: Pior Prognóstico no Acompanhamento de Dois Anos
Source: Arq Bras Cardiol. 2020 Apr 6;114(3):507–14. [Article in Portuguese] doi: 10.36660/abc.20190035 (PMC7792717; doi:10.36660/abc.20190035)
Supplement: Translation

# Translation: High Serum Netrin-1 and IL-1β in Elderly Females with ACS: Worse Prognosis in 2-years Follow-up

**Authors:** Paola Leocádio, Penélope Menta, Melissa Dias, Júlia Fraga, Alessandra Goulart, Itamar Santos, Paulo Lotufo, Isabela Bensenor, Jacqueline Alvarez-Leite

**Affiliations:** 1 BrazilDepartamento de Bioquímica e Imunologia - Universidade Federal de Minas Gerais, Belo Horizonte, MG – Brazil; 2 BrazilCentro de Pesquisa Clínica e Epidemiológica do Hospital Universitário da Universidade de São Paulo, São Paulo, SP – Brazil; 3 BrazilDepartamento de Clínica Médica da Faculdade de Medicina da Universidade de São Paulo, São Paulo, SP – Brazil

**Keywords:** Acute Coronary Syndrome/physiopathology, Netrin-1, Interleukin-1 beta, Atrial Remodeling, Hypertension, Diabetes Mellitus, Dyslipidemias, Stroke, Aged, Women

## Abstract

**Background:**

Several markers have been evaluated for a potential impact on clinical decisions or mortality prediction in acute coronary syndrome (ACS), including Netrin-1 and IL-1β that have been associated with cardiovascular disease.

**Objective:**

Our study examined the prognostic value of Netrin-1 and IL-1β in patients with ACS (2-year follow-up).

**Methods:**

We evaluate Netrin-1, IL-1β and other risk factors in the serum sample of 803 patients. Kaplan-Meier curves and Cox regression were used for the analysis of all-cause mortality, cardiovascular mortality, and a combined outcome of fatal myocardial infarction (MI) or new non-fatal MI, considering p-value < 0.05.

**Results:**

There were 115 deaths from all causes, 78 deaths due to cardiovascular causes and 67 events in combined outcomes. Netrin-1 levels above the median (>44.8 pg/mL) were associated with a worse prognosis (all-cause mortality and cardiovascular mortality) in elderly females, even after model adjustment (HR: 2.08, p = 0.038 and HR: 2.68, p = 0.036). IL-1β levels above the median (>13.4 pg/mL) in elderly females were associated with increased risk of all outcomes after adjustment (all-cause mortality - HR: 2.03, p = 0.031; cardiovascular mortality - HR: 3.01, p = 0.013; fatal MI or new non-fatal MI - HR: 3.05, p = 0.029). For males, no associations were observed between Netrin-1 or IL-1β and outcomes.

**Conclusion:**

High serum levels of Netrin-1 and IL-1β showed significant association with worse prognosis in elderly females. They may be useful as prognostic indicators in ACS. (Arq Bras Cardiol. 2020; 114(3):507-514)

## Introduction

Coronary heart disease (CHD) is the leading cause of death and years of life lost.^1^ Responsible for the largest number of deaths in Brazil, CHD has high prevalence and a poor prognosis.^2^ Despite the reduction in acute coronary syndrome (ACS) mortality observed in recent decades,^1^ it is estimated that near 14% of patients who have had a myocardial infarction (MI) will die of it.^3^ The risk of illness and death is 1.5 to 15 times higher for patients who survive the acute stage of MI than for the general population.^3^ Of those who have a first MI, approximately 17% of males and 21% of females at ≥ 45 years will have a recurrent MI or fatal CHD within five years.^3^

Inflammation is an important factor in the pathophysiology of ACS as well as in the cardiac remodeling after AMI. Several markers have been evaluated for a potential impact on clinical decisions or mortality prediction.^4^ Recently, neuronal guidance molecules, especially Netrin-1, have been identified as important modulators of atherosclerosis, although their specific role (protective or deleterious) is still controversial.^5-7^ Netrin-1 is a member of a family of proteins structurally similar to laminins, which are structural components of the basal membrane of tissues.^8^ The role of Netrin-1 in cardiovascular disease and inflammation is an emerging area of study.^5^

Interleukin-1 (IL-1) is one of the major mediators of inflammation-induced coagulation.^9^ IL-1β is capable of inducing the expression of other molecules that favor the recruitment of inflammatory cells to the lesion and tissue injury.^10^ A high level of IL-1 has been described in MI.^11,12^ Recently, a randomized clinical trial among people who suffered a MI showed that canakinumab, an agent that block IL-1β reduced the incidence of non-fatal CHD, non-fatal stroke events and cardiovascular death.^13^

Despite the importance of these two molecules in ACS, studies evaluating their prognostic value are scarce. Our objective was to evaluate the role of these molecules as predictors of prognosis in a 2-year follow-up.

## Methods

### Study design

The patients were participants of the “ERICO” (Strategy of Registry of Acute Coronary Syndrome) study, described in detail in previous reports.^14,15^ Briefly, it is a prospective cohort study that included individuals admitted to treatment for ACS at the University of São Paulo Hospital (HU-USP), a teaching community hospital with 260 beds located in the District of Butantan, São Paulo, Brazil, from February 2009 to December 2013. The study protocol was in accordance with the Declaration of Helsinki. This study was approved by the Research Ethics Committee (CEP-HU/USP 866/08), and all patients signed the Informed Consent Form.

Acute myocardial infarction (AMI) was defined by the presence of symptoms consistent with myocardial ischemia within 24 hours of hospital admission and troponin I level above the 99th percentile value with a coefficient of variation <10%. ST-segment elevation myocardial infarction (STEMI) was defined by the criteria for AMI, in addition to (a) the presence of persistent ST-segment elevation ≥1 mm in two contiguous electrocardiographic leads (lead ECG) or (b) new (or supposedly new) left bundle branch block (LBBB). Non-ST-segment elevation myocardial infarction (NSTEMI) was defined by the criteria for AMI plus the absence of persistent ST-segment elevation ≥1 mm in two contiguous ECG leads and of new or supposedly new LBBB. Unstable angina (UA) was defined as the presence of symptoms compatible with myocardial ischemia in the last 24 hours, absence of AMI diagnosis and at least one of the following five criteria: (a) history of previous coronary artery disease; (b) positive stratification of invasive or non-invasive ischemic heart disease; (c) dynamic or evolutionary ECG changes; (D) troponin I >0.4 ng/mL (ensuring troponin I levels above the 99th percentile regardless of the utilized kit) or (e) agreement on UA diagnosis between two independent physicians.

### Data collection and outcomes

After 6 months and annually for 2 years after the hospital admission, all individuals were contacted by telephone to update vital status information, including fatal and nonfatal cardiovascular outcomes. Whenever a participant reported a potential new MI event, new investigation procedures were initiated to confirm the event.

Study outcomes were all-cause mortality, cardiovascular mortality, and the combined outcome (fatal AMI and new non-fatal AMI). The strategy for collecting and classifying mortality data, including searching for official death records, was detailed in a previous report.^15^ In cases where it was not possible to determine the cause of death, the data were censored for all outcomes, except for death from all causes.

During the hospital phase, trained interviewers collected data related to sociodemographic characteristics, cardiovascular risk factors, and medication, as previously described.^15^ Blood samples were collected within 24 hours of admission. Analyses of plasma glucose, triglycerides, and total and HDL cholesterol were performed at HU-USP. LDL cholesterol was calculated using the Friedewald equation.^16^ Concentrations of Netrin-1 and IL-1β on admission were evaluated by Enzyme-Linked Immunosorbent Assay (ELISA), following the kit instructions (Netrin-1: SEB827HU; USCN Life Science Inc., Wuhan, China and IL-1β: 88-7010-88 eBioscience Inc., San Diego, CA, USA). Patients were classified according to Netrin-1 and IL-1β concentrations in “low” and “high” groups if their concentration were below or above the median.

### Statistical analysis

Data were assessed for normality using the Kolmogorov-Smirnov test. The chi-square and Mann-Whitney (all continuous variables presented nonparametric distribution) tests were used to compare groups. Values were expressed as median (interquartile interval) or n (%). Kaplan-Meier curves were used, and the log-rank test was used to evaluate the difference between low and high groups. Risk estimates (hazard ratios with their respective 95% confidence intervals) for the events were calculated using Cox regression. In addition to Netrin-1 and IL-1β, the following variables were used to construct models: age, type of ACS, diabetes, hypertension, and dyslipidemia. A two-tailed p-value < 0.05 was considered significant.

The software programs SPSS (IBM SPSS Statistics for Windows, version 22.0, Armonk, NY: IBM Corp.) and GraphPad Prism (version 5.01 for Windows, San Diego, California: GraphPad Software) were used to carry out the analyses.

## Results

A total of 803 patients were included in this study, including 333 women and 470 men. Comparing the main characteristics of male and female groups, we observed that women were older and had higher HDL-c concentration than men. Women were also more frequently affected by hypertension, diabetes, and dyslipidemia (Table 1). The most frequent type of ACS in male and female groups was NSTEMI (about 40% of cases) followed by UA and STEMI that had a similar frequency (about 30% each).

**Table 1:** – General characteristics in the hospital phase in males and females

Parameter	Male (n = 470)	Female (n = 333)	p
Age	60 (52 – 71)	65 (56 – 76)	< 0.0001
BMI	26.8 (23.8 – 29.6)	26.8 (24.0 – 30.9)	0.128
**ACS type**			**0.027**
NSTEMI	191 (40.6)	142 (42.6)	
STEMI	147 (31.3)	77 (23.1)	
UA	132 (28.1)	114 (34.2)	
**Smoking habits**			
Current	141 (31.3)	85 (27.2)	< 0.0001
Former	198 (43.9)	82 (26.3)	
Never	112 (24.8)	145 (46.5)	
Hypertension	339 (73.7)	267 (80.9)	0.018
Diabetes	156 (34.7)	148 (45.0)	0.004
Dyslipidemia	197 (48.0)	181 (60.7)	0.001
Glucose	125.0 (101.0 – 157.0)	124.0 (103.0 – 175.0)	0.652
Triacylglycerol	132.0 (94.0 – 190.3)	126.0 (97.0 – 183.0)	0.685
Total Cholesterol	171.5 (141.0 – 205.0)	170.0 (139.0 – 204.0)	0.720
HDL - Cholesterol	35.0 (30.0 – 44.0)	39.0 (32.0 – 46.5)	< 0.0001
LDL - Cholesterol	102.5 (77.0 – 134.3)	99.0 (77.0 – 124.3)	0.386
Netrin-1	44.8 (34.2 – 65.8)	44.8 (34.8 – 62.8)	0.813
IL-1β	15.1 (7.4 - 28.8)	13.8 (7.1 – 29.7)	0.536

Values are median (interquartile interval) or n (%). ACS: acute coronary syndrome. BMI: body mass index in kg/m^2^. HDL: high-density lipoprotein. LDL: low-density lipoprotein. NSTEMI: non-STsegment elevation Myocardial Infarction. STEMI: ST-segment elevation Myocardial Infarction. UA: unstable angina. Data of plasma glucose, triglyceridemia, total cholesterol, HDL and LDL are presented as mg/dL. Netrin-1 and IL-1β are presented as pg/mL. Mann-Whitney test or chi-square test. p-value comparing male and female groups.

During the 2 years follow up, there were 115 deaths from all causes (65 men and 50 women) including 78 deaths (67.8%) due to cardiovascular causes. We also identified 67 cases of AMI (fatal or non-fatal) in this same follow-up. Since age is an important factor involved in the mortality rate, we analyzed separately in women and men younger and older than 60 years.

To evaluate a possible role of Netrin-1 and IL-1β as prognostic markers, we compared the frequency of ACS in the patients with levels of Netrin-1 and IL-1β levels above and below the respective median. There were no associations between levels of Netrin-1 and IL-1β and all-cause mortality, cardiovascular mortality and fatal or new non-fatal MI outcomes for males independently of their age (data not shown). For this reason, we focused our investigation on the female group (333 patients). The main characteristics of the female group (younger and older than 60 years) are shown in Table 2.

**Table 2:** – General characteristics in the hospital phase in younger (<60 y) and older (>60 years) females

Parameter	Total (n = 333)	Younger (<60 years) (n = 111)	Older (>60 years) (n = 222)	p
BMI	26.8 (24.0 – 30.9)	26.8 (24.5 - 31.1)	26.7 (23.8 – 30.8)	0.532
**ACS type**				**0.031**
NSTEMI	142 (42.6)	40 (36.0)	102 (45.9)	
STEMI	77 (23.1)	35 (31.5)	42 (18.9)	
UA	114 (34.2)	36 (32.4)	78 (35.1)	
**Smoking habits**				**< 0.0001**
Current	85 (27.2)	47 (44.3)	38 (18.4)	
Former	82 (26.3)	28 (26.4)	54 (26.2)	
Never	145 (46.5)	31 (29.2)	114 (55.3)	
Hypertension	267 (80.9)	79 (72.5)	188 (85.1)	0.006
Diabetes	148 (45.0)	39 (35.8)	109 (49.5)	0.018
Dyslipidemia	181 (60.7)	48 (48.5)	133 (66.8)	0.002
Glucose	124.0 (103.0 – 175.0)	121.0 (103.0 – 163.0)	128.0 (104.0 – 180.0)	0.381
Triacylglycerol	126.0 (97.0 – 183.0)	151.0 (97.0 – 201.0)	122.0 (93.5 – 174.0)	0.080
Total Cholesterol	170.0 (139.0 – 204.0)	185.0 (157.5 – 215.3)	161.0 (134.0 – 196.0)	0.002
HDL- Cholesterol	39.0 (32.0 – 46.5)	36.0 (32.0 – 45.0)	41.0 (33.0 – 47.2)	0.065
LDL- Cholesterol	99.0 (77.0 – 124.3)	114.0 (89.0 – 134.0)	93.0 (72.0 – 118.0)	0.0004
Netrin-1	44.8 (34.8 – 62.8)	44.8 (33.8 – 65.8)	44.8 (34.7 – 65.0)	0.861
IL-1β	13.8 (7.1 – 29.7)	15.5 (7.9 – 49.7)	13.4 (7.1 – 24.1)	0.037

Values are median (interquartile interval) or n (%). ACS: acute coronary syndrome. BMI: body mass index in kg/m^2^. HDL: high-density lipoprotein. IL-1β: Interleukin - 1beta. LDL: low-density lipoprotein. NSTEMI: non-STsegment elevation Myocardial Infarction. STEMI: ST-segment elevation Myocardial Infarction. UA: unstable angina. Data of plasma glucose, triacylglycerol, total cholesterol, HDL and LDL are presented as mg/dL. Netrin-1 and IL-1β are presented as pg/mL. Mann-Whitney test or chi-square test.

At admission, women presented similar values of BMI, serum concentrations of glucose, triacylglycerol, and HDL cholesterol regardless in both age groups. The frequency of important risk factors such as hypertension, dyslipidemia and diabetes were higher in older women. However, levels of LDL cholesterol were lower in older ones. Current smokers were more frequent in younger women while more than 50% of the older women never smoked (Table 2). We did not find differences in the median of Netrin-1 between age groups. However, the median of IL-1β was higher in the younger group.

Associations between low and high Netrin-1 or IL1- β and the outcomes, according to the age range were presented in Table 3 and Table 4, respectively. The number of death of all-cause was very low (3 death) in the women younger than 60 years and only 6 cases of the combined outcome, avoiding reliable analyzes in this group. However, in the older (>60 years) group, we found associations between the highest level of Netrin-1 and deaths from all causes and cardiovascular causes. An association between high IL-1β and death for CVD as also found among older women (p = 0.034).

**Table 3:** – Outcomes according to the Netrin-1 in females

	Female - Total			< 60 years			> 60 years		
2 years follow-up	Low Netrin-1	High Netrin-1	p	Low Netrin-1	High Netrin-1	p	Low Netrin-1	High Netrin-1	p
All-cause mortality	18 (36.0)	32 (64.0)	0.021	2 (66.7)	1 (33.3)	0.612	16 (34.0)	31 (66.0)	0.011
Cardiovascular mortality	12 (35.3)	22 (64.7)	0.052	2 (66.7)	1 (33.3)	0.612	10 (32.3)	21 (67.7)	0.029
Fatal or new non-fatal MI	11 (39.3)	17 (60.7)	0.193	4 (66.7)	2 (33.3)	0.467	7 (31.8)	15 (68.2)	0.066

Values are n (%). MI: myocardial infarction. Chi-square test.

**Table 4:** – Outcomes according to the IL-1β in females

	Female - Total			< 60 years			> 60 years		
2 years follow-up	Low IL-1β	High IL-1β	p	Low IL-1β	High IL-1β	p	Low IL-1β	High IL-1β	p
All-cause mortality	21 (42.0)	29 (58.0)	0.221	0 (0.0)	3 (100.0)	0.118	19 (40.4)	28 (59.6)	0.140
Cardiovascular mortality	12 (35.3)	22 (64.7)	0.071	0 (0.0)	3 (100.0)	0.118	10 (32.3)	21 (67.7)	0.034
Fatal or new non-fatal MI	10 (35.7)	18 (64.3)	0.117	2 (33.3)	4 (66.7)	0.438	7 (31.8)	15 (68.2)	0.075

Values are n (%). IL-1β: Interleukin - 1beta. MI: myocardial infarction. Chi-square test.

These data showing a worse prognostic in older females with high levels of Netrin-1 and IL-1β at admission were confirmed by Kaplan-Meier curves. High levels of Netrin-1 showed a lower rate of survival when considering all-cause mortality (p = 0.011, Figure 1A) and also considering only cardiovascular deaths (p = 0.024, Figure 1B). The marker only tended to be associated with fatal MI or new non-fatal MI (p = 0.067, Figure 1C). High levels of IL-1β also showed a lower rate of survival when considering cardiovascular deaths (p = 0.031, Figure 1E) and tended to be associated with fatal MI or new non-fatal MI (p = 0.064, Figure 1F).

**Figure 1:** – Kaplan-Meier curves for Netrin-1 (A – C) and IL-1Β (D – F) in older (>60 years) females considering a 2-year follow-up. IL-1β: Interleukin - 1beta. MI: myocardial infarction.

The analysis of the hazard ratios (Table 5) showed an increased risk of death from all causes for the high Netrin-1 group that remained significant in the adjusted model. The same results were seen for risk of death from cardiovascular causes.

**Table 5:** – Hazard Ratios for high Netrin-1 and IL-1β and all-cause mortality, cardiovascular mortality and fatal or new non-fatal MI in older (>60 years) females

	All-cause mortality		Cardiovascular mortality		Fatal or new non-fatal MI	
	HR (95% CI)	p	HR (95% CI)	p	HR (95% CI)	p
	**Netrin-1**					
Crude	2.15 (1.17 – 3.93)	0.013	2.31 (1.09 – 4.92)	0.029	2.25 (0.92 – 5.54)	0.075
Adjusted *	2.08 (1.04 – 4.16)	0.038	2.68 (1.06 – 6.74)	0.036	1.82 (0.65 – 5.07)	0.247
	**IL-1β**					
Crude	1.57 (0.87 – 2.81)	0.127	2.23 (1.05 – 4.75)	0.036	2.27 (0.92 – 5.58)	0.073
Adjusted *	2.03 (1.06 – 3.89)	0.031	3.01 (1.26 – 7.17)	0.013	3.05 (1.12 – 8.32)	0.029

CI: confidence interval. HR: hazard ratio. IL-1β: Interleukin - 1beta. MI: myocardial infarction. * Adjusted for age, type of Acute Coronary Syndrome, diabetes, hypertension and dyslipidemia.

Considering the high levels of IL-1β, we did not find significant HR in the crude model for all-cause mortality and fatal or new non-fatal MI (Table 5). However, significant HR for all-cause mortality and fatal or new non-fatal MI were observed in the adjusted model. We also observed an increased risk of death from cardiovascular causes for the high IL-1β group even after model adjustment.

## Discussion

This work is the pioneer in evaluating the prognostic value of Netrin-1 in ACS and presents new information about the prognostic value of IL-1β in this condition.

In our study, we observed an association between the highest levels of Netrin-1 and worse prognosis when all-cause mortality and cardiovascular mortality were analyzed, in elderly females.

Plasma Netrin-1 is a diagnostic biomarker of many cancer types.^17-19^It was verified that the higher gene expression or concentration of Netrin-1 in these tissues was associated with a worse prognosis, probably related to the anti-apoptotic and angiogenic effects of Netrin-1. However, the role of Netrin-1 in atherosclerosis and cardiac remodeling after MI is still controversial. It has been described that netrin-1 could promote or protect against atherosclerosis, in the dependency of environmental conditions.^20^ Reduced endogenous levels of Netrin-1 can also lead to deleterious effects since pro-atherogenic factors can reduce the expression of this molecule.^20^ In models of MI, netrin-1 administration reduced the severity of myocardium lesion when compared to the non-supplemented controls.^21,22^

In our study, it is possible that the "High Netrin-1" group is composed of patients who had a more severe ACS event. Our hypothesis is that the level of Netrin-1 increases in more severe cases of ACS. This hypothesis is based on studies that indicate that the expression of Netrin-1 is induced after cellular injury and can be used as a biomarker for organ damage or disease^23^ as seen in the cardiac surgery.^24^ Moreover, hypoxia, a condition closely linked to atherosclerosis and ACS is also an inducer of Netrin-1 expression.^25^ Moreover, Van Gils et al.^6^ observed increased expression of the molecule in cholesterol-loaded macrophages promoting the retention of these cells *in vitro*, which could contribute to a more rapid evolution of the atherosclerotic plaque and consequently increase the chance of thrombus formation and occurrence of infarction.

These factors lead us to believe that the highest number of unfavorable outcomes, as well as the higher risk of death from all causes and from cardiovascular causes observed in women over 60 years of age with higher Netrin-1 levels are associated with the severity of event and the higher degree of inflammation (as suggested by the high levels of IL-1β) which may have contributed to a worse prognosis.

Regarding IL-1β, high levels of this one and other pro-inflammatory cytokines have already been identified in patients with ACS.^26^ Nonetheless, few studies were addressed to the prognostic value of this cytokine.^27,28^ In agreement with our results, these studies suggest that higher levels of IL-1β were seen in patients with ACS who underwent new events during follow-up.

IL-1β is capable of increasing the expression of molecules that contribute to plaque rupture and thrombus formation, culminating in the occurrence of ACS.^10,29^ Thus, higher levels of serum IL-1β could reflect exacerbated inflammation, favoring the occurrence of cardiovascular complications.

Higher IL-1β levels suggest an exacerbated inflammation that could impair cardiac remodeling. Adverse remodeling after MI is the structural basis for ischemic heart failure. Although adequate amounts of IL-1β and other inflammatory cytokines are essential in the initial phase of remodeling, the decrease of cytokine levels is needed to promote effective healing.^30^ It has been described that elevated levels of IL-1β up to two months after infarction in patients with STEMI were strongly associated with a worsening of cardiac function after one year of follow-up.^31^ Furthermore, cytokine was a strong predictor of left ventricular hypertrophy, which is important in predicting cardiovascular morbidity and mortality.^31^

Several factors may help to understand the absence of association with worse prognosis found among men and younger women. Cardiac and vascular tissues are influenced by hormones such as estrogen and testosterone, varying according to sex and age.^32,33^ Older women have a larger left ventricle mass than men, due to factors that indicate lower arterial capacity, such as reduced carotid wall thickness.^33^

The relationship between inflammatory cytokines and gender has not yet been elucidated, although differences between concentrations are observed in the literature.^32^ Studies indicate that IL-β concentration is higher in males.^33,34^ Furthermore, cytokine levels are inversely related to age, as seen in our work and literature.^35^ The literature does not provide data on sex differences for Netrin-1. Since we did not observe statistical gender differences in the levels of the markers, our hypothesis is that in our female group the levels of these were higher than the normal. This possible increase may be related to the higher frequency of cardiovascular risk factors in this group in the present study, factors that may lead to an increase in levels of inflammatory markers.^36^ However, even after adjusting for these factors, high levels of Netrin-1 and IL-1β remained associated with worse prognosis, demonstrating that these inflammatory markers are independently associated with worse prognosis, and may be related to the reduction of arterial capacity already observed in older women when compared to men.

We point out as limitations of the study its unicentric characteristic and the absence of a control group. Furthermore, we did not collect data from the pre-event period, which would allow us to determine the variation in marker concentration after ACS.

## Conclusions

Higher levels of Netrin-1 and IL-1β are associated with worse prognosis in elderly females with ACS. The mechanism for such association can be related to maintenance of inflammation and adverse cardiac remodeling, propitiating further cardiovascular events.
